# Preparation and Space Charge Properties of Functionalized Zeolite/Crosslinked Polyethylene Composites with High Thermal Conductivity

**DOI:** 10.3390/polym15224363

**Published:** 2023-11-09

**Authors:** Bai Han, Jinghui Dai, Wanliang Zhao, Wei Song, Zhi Sun, Xuan Wang

**Affiliations:** 1 Key Laboratory of Engineering Dielectrics and Its Application, Ministry of Education, Harbin University of Science and Technology, Harbin 150080, China; zliang1997@163.com (W.Z.); songwei791214@163.com (W.S.); sunzhimems@163.com (Z.S.); wangxuan@hrbust.edu.cn (X.W.); 2 College of Electrical and Electronic Engineer, Harbin University of Science and Technology, Harbin 150080, China

**Keywords:** crosslinked polyethylene, zeolite, thermal conductivity, dielectric properties, space charge

## Abstract

Nanocomposite doping is an effective method to improve the dielectric properties of polyethylene. Meanwhile, the introduction of thermal conductivity groups in crosslinked polyethylene (XLPE) is also an effective way to improve the thermal conductivity. Nano-zeolite is an inorganic material with a porous structure that can be doped into polyethylene to improve the insulation performance. In this paper, hyperbranched polyarylamide (HBP) with a high thermal conductivity and an auxiliary crosslinking agent (TAIC) was grafted on the surface of ZSM-5 nano-zeolite successively to obtain functionalized nano-zeolite (TAICS-ZSM-5-HBP) (the “S” in TAICS means plural). The prepared functionalized nano-zeolite was doped in polyethylene and grafted under a thermal crosslinking reaction to prepare nanocomposites (XLPE/TAICS-ZSM-5-HBP). The structural characterization showed that the nanocomposite was successfully prepared and that the nanoparticles were uniformly dispersed in the polyethylene matrix. The space charge of the TAICS-ZSM-5-HBP 5wt% nanocomposite under a high electric field was obviously inhibited. The space charge short-circuit test showed that the porous structure of the nano-zeolite introduced more deep traps, which made the trapped charge difficult to break off, hindering the charge injection. The introduction of TAICS-ZSM-5-HBP particles can greatly improve the thermal conductivity of nanocomposites. The thermal conductivity of the XLPE/5wt% and XLPE/7wt% TAICS-ZSM-5-HBP nanocomposites increased by 42.21% and 69.59% compared to that of XLPE at 20 °C, and by 34.27% and 62.83% at 80 °C.

## 1. Introduction

The rapid development and popularization of new-energy power-generation technology has put forward higher requirements for the stability and performance of high-voltage direct current (HVDC) transmission systems for long-distance transmission. Crosslinked polyethylene (XLPE), as a relatively mainstream insulating material, is widely used in high-voltage cables. However, the thermal conductivity of ordinary XLPE cables is low, and it is easy to cause the aging and damage of XLPE-insulated cables in high-temperature and high-electric-field working environments. Moreover, the accumulation of space charge in the insulation material of HVDC cables causes electric field distortion, resulting in insulation failure. These factors drastically reduce the service life of cables and limit the development of high-voltage DC cables [1,2,3].

Many studies have found that nanoparticle-doped polymers are a feasible method to improve the performance of dielectric materials, and they have always been a hot spot in the field of engineering dielectrics. However, many nanoparticles are poorly dispersed in polyethylene, and the agglomeration of nanoparticles will introduce large size defects, leading to a deterioration in the performance of the material. Therefore, the effectiveness of a composite modification of nanoparticles largely depends on the dispersion of the nanoparticles [4,5,6,7,8,9].

Nano-zeolite has a special porous structure, and the specific surface area is much higher than ordinary nano-oxide particles, which can greatly enhance the interface effect in nano-dielectric materials. Therefore, a doping modification using nano-zeolite has a good prospect to improve the dielectric properties of polyethylene [10,11]. However, the surface energy of nano-zeolite particles is also high. The compatibility between nano-zeolite and polyethylene matrix material is poor, and the particle agglomeration phenomenon easily occurs when nano-zeolite is doped. Therefore, it is necessary to use coupling agents to organically modify the surface of the nano-zeolite and improve its dispersion performance in the polyethylene matrix. At the same time, other functional groups, such as groups improving thermal conductivity or auxiliary crosslinking agents, can be introduced by grafting to make the nano-zeolite more evenly dispersed and fixed in the XLPE crosslinking network [12,13,14].

In this paper, the surface of ZSM-5 zeolite was modified and grafted with hyperbranched polyarylamide (HBP) and functionalized using a crosslinking agent (TAIC). The TAICS-ZSM-5-HBP zeolite was introduced into the cross-network structure of XLPE and nanocomposites of XLPE/TAICS-ZSM-5-HBP were prepared using a crosslinking reaction. Nano-zeolite doping gave full play to the surface effect and small-quantum-size effect of nanoparticles in nano-dielectric materials (TAICS means the plural form of TAIC). Meanwhile, the huge specific surface area brought by the porous structure of nano-zeolite was used to increase the interface region size and enhance the interface effect, which effectively improved the dielectric properties of the XLPE composites. Through surface modification and grafting, high-thermal-conductivity groups and auxiliary crosslinking agents were introduced to improve the dispersion of the nanoparticles and the thermal conductivity of the nanocomposites.

## 2. Materials and Methods

### 2.1. Material Preparation

**Figure 1:**
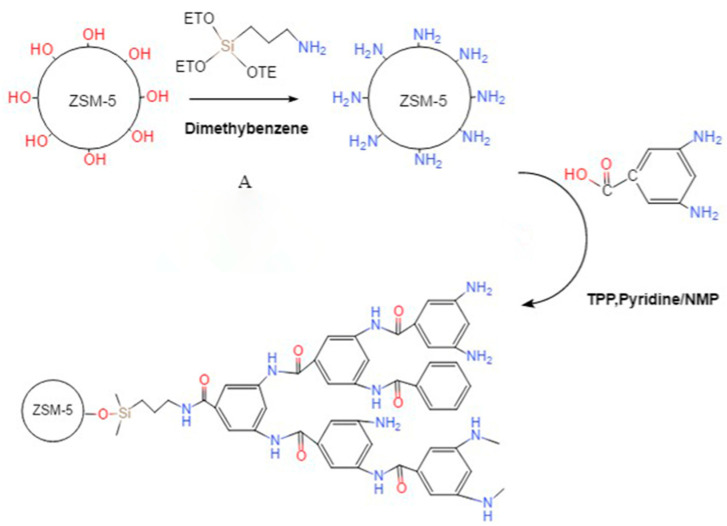
Schematic diagram of the preparation of ZSM-5-HBP.

First, the surface modification of nano-ZSM-5 zeolite (Nankai University Catalyst Co., Ltd., Tianjin, China) was performed and the hyperbranched polyacrylamide (HBP) was subsequently grafted to the surface of the ZSM-5 zeolite. The chemical reaction process and chemical structure of ZSM-5-HBP are shown in Figure 1. The preparation principle is as follows: In order to introduce amino functional groups on the surface of ZSM-5 zeolite, it was necessary to make a basis modification for subsequent overbranching. Solution A was prepared with absolute ethanol:γ-aminopropyltriethoxysilane (KH550) (Shanghai Macklin Biochemical Technology Co., Ltd., Shanghai, China):distilled water in a ratio of 7:2:1 and treated with an ultrasonic shock for 30 min. An amount of 10 g of type ZSM-5 zeolite particles was dispersed into 250 mL of absolute ethanol, and the solution was mixed well in a three-neck flask. Solution A was added to a three-neck flask and reacted by magnetically stirring for 6 h at a temperature of 65 °C and in a nitrogen atmosphere. After centrifugation, repeated washing (the washing liquid was absolute ethanol), and vacuum drying, amino-functionalized ZSM-5 zeolite was obtained.

Hyperbranched polyarylamides (HBPs) with a rigid backbone, which improved the thermal conductivity, were grafted onto the amino-functionalized zeolite ZSM-5 surface [15]. The specific preparation process is as follows: 6.5 g of amino-functionalized ZSM-5-type zeolite, 0.65 g of 3,5-diaminobenzoic acid(Shanghai Macklin Biochemical Technology Co., Ltd., Shanghai, China), 6 mL of pyridine(Aladdin Scientific Co., Ltd., Shanghai, China), and 6 mL of triphenyl phosphite(Tianjin Kemiou Chemical Reagent Co., Ltd., Tianjin, China) were dispersed into 200 mL of an *N-*methylpyrrolidone (Tianjin Kemiou Chemical Reagent Co., Ltd., Tianjin, China) solution. A magnetic stirring reaction was performed under a temperature of 80 °C and a nitrogen atmosphere for 3 h. After centrifugation, repeated washing (the washing solution was methanol and *N,N*-dimethylformamide), and vacuum drying, ZSM-5 zeolite with surface-grafted HBP (ZSM-5-HBP) was obtained.

**Figure 2:**
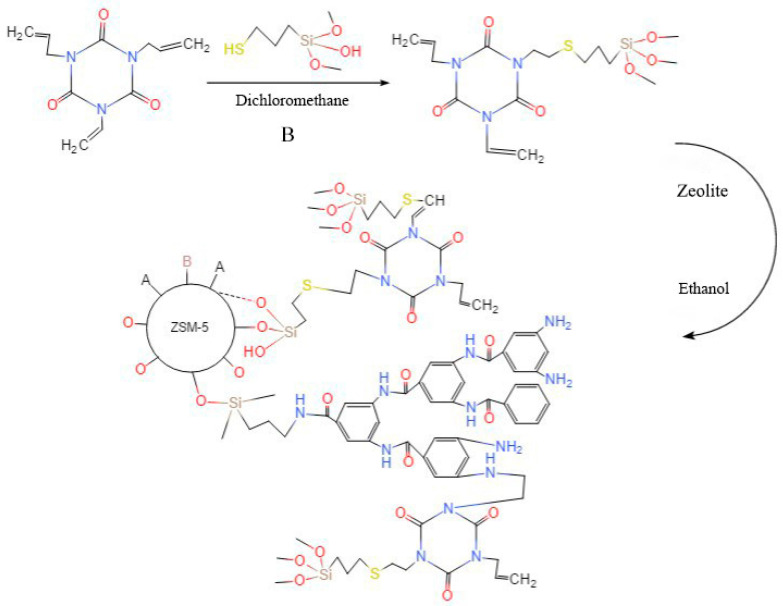
Schematic diagram of the preparation of TAICS-ZSM-5-HBP.

Next, the auxiliary crosslinker was grafted onto ZSM-5-HBP. Based on the thiol-double bond Michael addition reaction, the auxiliary crosslinker was treated with a silane coupling agent and grafted on the surface of the binder. The preparation reaction is shown in Figure 2, and the experimental content is as follows:

An amount of 6.5 g of trihydroxypropyl isocyanurate (TAIC) (Shanghai Macklin Biochemical Technology Co., Ltd., Shanghai, China) and 10 mL of dichloromethane (DCM) (Tianjin Fuyu Fine Chemical Co., Ltd., Tianjin, China) were added to a three-neck flask, and ultrasonic shaking was applied for 10 min. A total of 3 g of 3-mercaptopropyltrimethoxysilane (KH590) (Shanghai Macklin Biochemical Technology Co., Ltd., Shanghai, China), 0.3 g of triethylamine (TEA) (Tianjin Fuyu Fine Chemical Co., Ltd., Tianjin, China), and 6 ml of DCM were mixed well to obtain solution B. Solution B was slowly dropped into the above three-neck flask under a nitrogen atmosphere. The mixture was reacted at room temperature for 18 h to obtain the liquid intermediate TAICS.

A total of 6.5 g of vacuum-dried ZSM-5-HBP was dispersed into 100 mL of absolute ethanol:water = 4:1 solution. After mixing well with ultrasonic shaking for 5 min at room temperature, its pH was adjusted to about 4 with a hydrochloric acid solution. Then, the mixed solution was transferred to a beaker and 15 mL of TAICS was dropped into it; the mixture was reacted for 80 h under magnetic stirring at a 65 °C temperature and in a nitrogen atmosphere. After centrifugation, repeated washing (the washing liquid was absolute ethanol), and vacuum drying, zeolite (TAICS-ZSM-5-HBP) with assisted crosslinking functionalization and surface-grafted hyperbranched polyarylamide was obtained.

In order to form a comparative experiment, XLPE, XLPE/5wt% ZSM-5, XLPE/5wt% TAICS-ZSM-5-HBP, and XLPE/7wt% TAICS-ZSM-5-HBP composites were prepared. The materials required for preparation and the specific preparation process are as follows:

TAICS-ZSM-5-HBP modified particles or ZSM-5 particles were dispersed in an ethanol solution with LDPE according to the desired mass fraction. After 30 min of ultrasonic shaking, the solution was placed in an 80 °C vacuum-drying oven for 6 h. The torque rheometer temperature was set to 120 °C and the speed was set to 60 rpm, and when the torque rheometer temperature reached the set value, the mixed LDPE was added to the silo for melting. After 10 min, the remaining required crosslinker DCP and TAIC were added in the prescribed proportion. Mixing was continued for 10 min, and then the composite material was removed and cut into pellets for later use.

According to the requirements of each test, the pellets prepared in the previous step were taken and weighed. The pellets were placed in a plate vulcanizer and melted at 120 °C for 5 min; the vulcanizer was gradually pressurized to 15 MPa, the temperature continued to rise to 175 °C, and 15 MPa was maintained for 30 min to complete the crosslinking reaction. Nanocomposite materials (XLPE, XLPE/5wt% ZSM-5, XLPE/5wt% TAICS-ZSM-5-HBP, and XLPE/7wt% TAICS-ZSM-5-HBP) were prepared. Before the following test, the experimental specimen was placed in a vacuum-drying oven at 60 °C for degassing and a short-circuit treatment for 24 h.

### 2.2. SEM Measurement

The appearance and surface structure of the sample, such as the particle size, surface morphology, and dispersion, were directly observed using scanning electron microscopy (SEM) [16]. Before the SEM test, the sample with a thickness of 300 µm was broken to obtain a fracture surface in liquid nitrogen, and the fracture surface of the sample was gold-plated using an E-1045 Ion Sputter instrument (Hitachi High-Tech Co., Ltd., Tokyo, Japan) to improve the electrical conductivity of the sample. The sample was subsequently observed using SEM equipment. The morphology of the nanocomposite materials (XLPE, XLPE/5wt% ZSM-5, XLPE/5wt% TAICS-ZSM-5-HBP, and XLPE/7wt% TAICS-ZSM-5-HBP) was characterized using a Hitachi SU8020 SEM (Hitachi High-Tech Co., Ltd., Tokyo, Japan) at an accelerating voltage of 10 kV.

### 2.3. FTIR Measurement

Fourier-transform infrared spectroscopy is a method that combines the Fourier transform and infrared spectroscopy. Infrared spectroscopy obtained using Fourier-transform technology can identify functional groups of chemicals [17]. The chemical structures of ZSM-5 zeolite, ZSM-5-HBP modified particles, TAICS intermediate products, and TAICS-ZSM-5-HBP modified particles were characterized using Fourier-transform infrared spectroscopy on a JASCO FT/IR-6100 spectrometer (JASCO Corporation, Tokyo, Japan), and the characteristic peaks of each group were measured and compared to verify the chemical reaction. Before the measurement, the powder was dried to a constant weight under a vacuum-drying box. Subsequently, potassium bromide and the sample powder were mixed evenly (potassium bromide:zeolite = 100:1), ground in a mortar, and then pressed into a tablet. The infrared spectrum was measured from 4000 cm^−1^ to 400 cm^−1^ with a spectral resolution of 2 cm^−1^ using the reflection mode. The infrared absorption spectrum was tested 10 times and the results were averaged.

### 2.4. Space Charge Measurement

**Figure 3:**
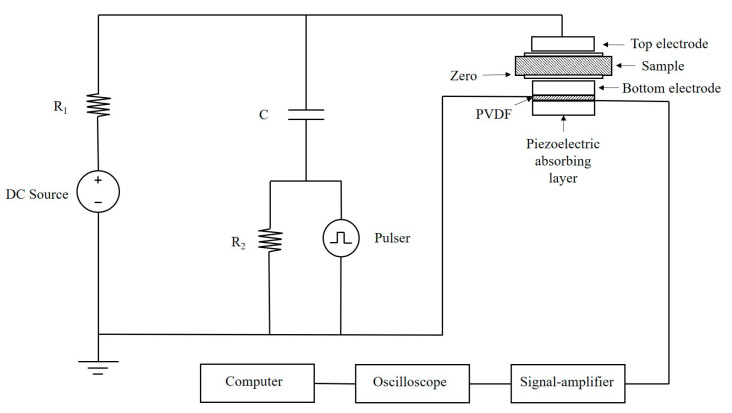
Installation diagram of space charge test system.

The space charge measurement was performed in a pulsed electro-acoustic (PEA) system (self-made device) [18]. By analyzing the process of pressure signal propagation and superposition, the obtained electrical signal waveform was processed to obtain the vertical distribution of charge in the sample. When the electric field was applied for a predetermined time, the high voltage source could be removed so that both sides of the sample were grounded. The space charge distribution at different short-circuit times could be detected. The simplified diagram of the experimental device is shown in Figure 3. Before the PEA test, degassing and short-circuit pretreatment were carried out in a vacuum oven at 60 °C. The equipment parameters were set as follows: pulse amplitude, 500 V; width, 10 ns; polarization electric field, 40 kV/mm for 3600 s; and short-circuit depolarization for 1800 s at different temperatures of 25 °C and 80 °C.

### 2.5. Thermal Conductivity Measurement

The thermal conductivity of the nanocomposites was measured at different temperatures using an LFA 447 laser thermal conductivity analyzer (NETZSCH Corporation, Selb, Germany). The laser flash method used by the laser thermal conductivity analyzer relied on the laser source to instantly emit a pulsed beam as a heat source to heat the sample, and the infrared detector was used to detect the heat conducted on the back of the sample. Then, the thermal diffusion coefficient and thermal conductivity were analyzed. Before the thermal conductivity test on all the samples, the graphite coating should be uniformly sprayed on both sides of the test sample to reduce the reflection of laser pulses and improve the absorption of laser pulses on the surface of the specimen, which reduces the heat loss and the calculation error. The size of the sample was strictly specified as a circular film with a diameter of 25.4 mm, and the test temperature was measured in the range of 20–80 °C at intervals of 10 °C.

## 3. Results and Discussion

**Figure 4:**
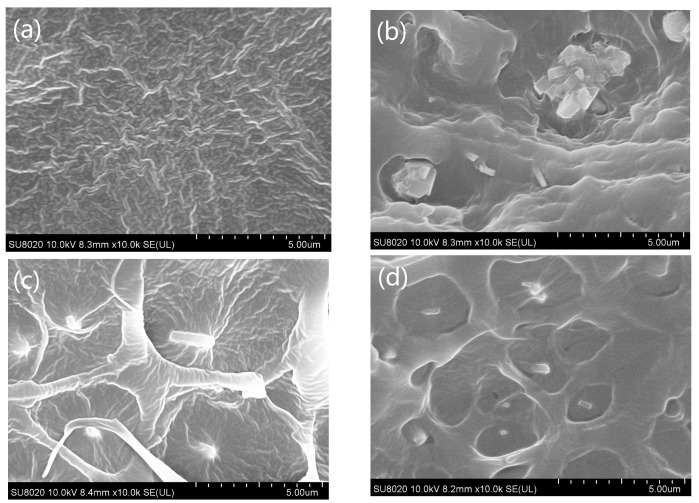
SEM images of XLPE and nanocomposite materials: (**a**) XLPE, (**b**) XLPE/5wt%/ZSM-5, (**c**) XLPE/5wt% TAICS-ZSM-5-HBP, and (**d**) XLPE/7wt%-TAICS-ZSM-5-HBP.

The XLPE, XLPE/5wt% ZSM-5, XLPE/5wt% TAICS-ZSM-5-HBP, and XLPE/7wt% TAICS-ZSM-5-HBP material fracture surface scanning electron microscopy diagrams are shown in Figure 4.

Figure 4a is the fracture surface SEM image of crosslinked polyethylene without any dopants. It can be seen from Figure 4b that the ZSM-5 zeolite particles without any modification in the XLPE/5wt% ZSM-5 composite were seriously agglomerated, and the size of the agglomerated individuals was very large. The XLPE/5wt% TAICS-ZSM-5-HBP and XLPE/7wt% TAICS-ZSM-5-HBP composites had no obvious agglomeration phenomenon, and the particles were uniformly dispersed, as shown in Figure 4c,d. This was mainly due to the untreated ZSM-5-type zeolite particles having a higher surface energy, making it easier for them to agglomerate. However, the modified zeolite particles not only had a lower surface energy and did not easily agglomerate after surface modification, but they could also be directly grafted on the polyethylene macromolecular chain to participate in the crosslinking reaction after crosslinking functionalization and grafting HBP, which improved the compatibility between the modified particles and XLPE and made the modified particles disperse more evenly.

**Figure 5:**
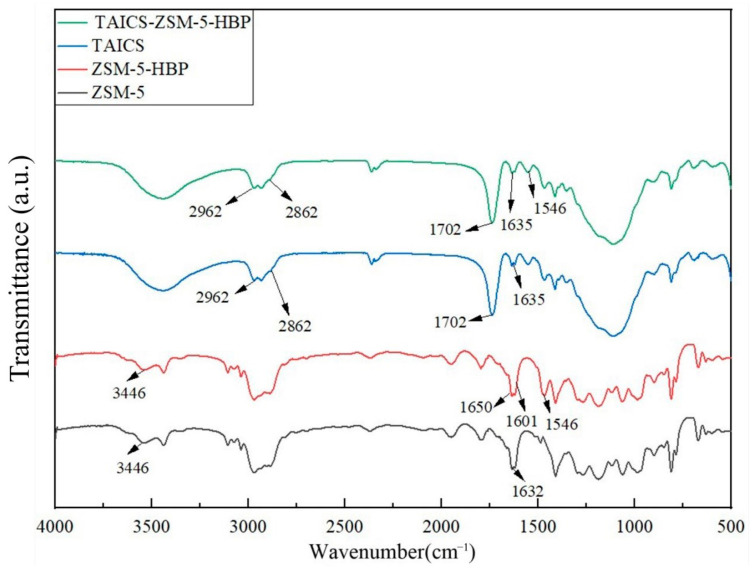
Infrared spectra of ZSM-5, ZSM-5-HBP, TAICS, and TAICS-ZSM-5-HBP.

The chemical structure of the ZSM-5 zeolite, ZSM-5-HBP modified particles, TAICS intermediate products, and TAICS-ZSM-5-HBP modified particles and the characteristic peaks of their groups were measured for comparison to verify the progress of the chemical reaction. The measured infrared spectra are shown in Figure 5.

**Figure 6:**
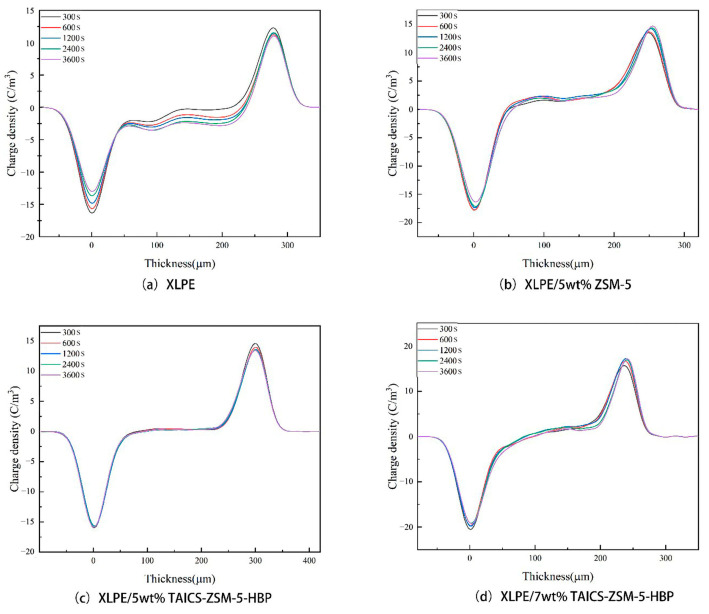
Space charge distribution of XLPE and its composite during pressurization at 25 °C.

**Figure 7:**
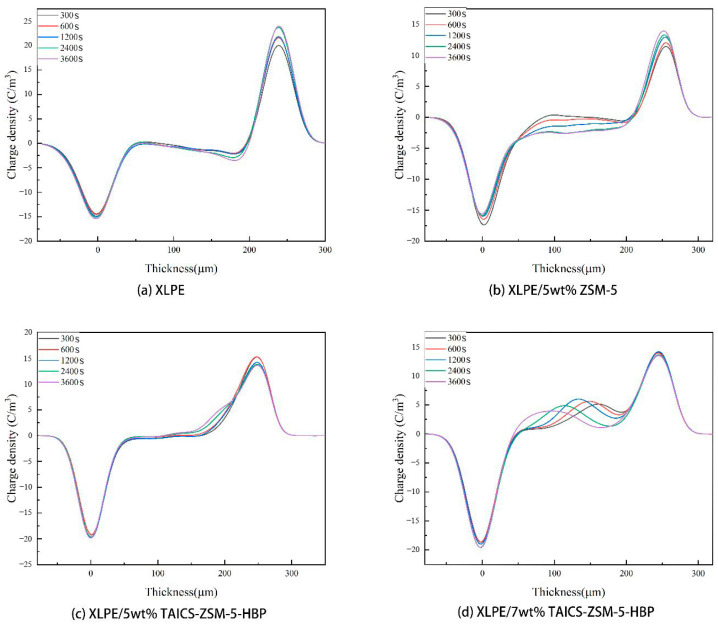
Space charge distribution of XLPE and its composite during pressurization at 80 °C.

It can be seen from Figure 5 that the absorption peak of ZSM-5 zeolite at 3446 cm^−1^ was the characteristic peak of the Si-OH group, and the absorption peak at 1632 cm^−1^ was the bending vibration peak of the Si-OH group. Compared with the infrared spectra of ZSM-5 zeolite, ZSM-5-HBP had stretching peaks of the -NHCO group at 1546 cm^−1^ and 1650 cm^−1^ and a characteristic peak of a benzene ring at 1601 cm^−1^, which indicated that the hyperbranched polyacrylamide (HBP) was successfully grafted onto ZSM-5 zeolite. At the same time, the absorption peak of the ZSM-5 at 1632 cm^−1^ was significantly weakened, indicating that the -OH group on the surface of ZSM-5 was reduced, and that an -NH2 group was formed by amination during the surface modification process and then grafted with HBP. From the infrared spectrum of the intermediate product TAICS, a double bond stretching vibration peak of the -CH=CH_2_ group at 1635 cm^−1^, an absorption peak of the -CO group at 1702 cm^−1^, and absorption peaks of the -CH_3_ group at 2862 cm^−1^ and 2961 cm^−1^ were found, confirming the successful preparation of the TAICS intermediate. At the same time, TAICS-ZSM-5-HBP also had absorption peaks at 1635 cm^−1^, 1702 cm^−1^, 2862 cm^−1^, and 2961 cm^−1^, indicating that the intermediate product existed in the modified particles of TAICS-ZSM-5-HBP. This confirmed the successful grafting of TAICS onto the ZSM-5-HBP modified particles. The above results show that the TAICS-ZSM-5-HBP modified zeolite with assisted crosslinking functionalization and surface grafting of HBP was successfully prepared. The charge density distribution of the composites at different temperatures was compared. The effects of the space charge behavior and space charge accumulation on the internal electric field distribution of the composites are discussed. The space charge distribution of the XLPE, XLPE/5wt%ZSM-5 composites, XLPE/5wt%TAICS-ZSM-5-HBP composites, and XLPE/7wt%TAICS-ZSM-5-HBP composites during pressurization were tested at 25 °C and 80 °C, and the results are shown in Figure 6 and Figure 7.

It can be seen from Figure 6a that, at a temperature of 25 °C and a high electric field of 40 kV/mm, a small amount of heteropolar charge began to accumulate around the positive and negative electrodes of the XLPE at the beginning of the voltage application, and the charge density increased with an increase in the polarization time. This is because the molecular chains inside the XLPE had some free space, and electrons could easily overcome the repulsion barrier between the molecular chains to reach the anode and become caught by traps in the XLPE. Impurities present in the XLPE could also dissociate the anion and cation, which moved in the opposite direction of their polarity, so that the internal charge of the material accumulated.

As shown in Figure 6b, compared with XLPE, the internal space charge density of the XLPE/5wt% ZSM-5 composite decreased to a certain extent, which was due to the introduction of a large number of deep-energy-level traps by zeolite particles in the composite. A trap near the electrode in the sample captured the charge injected by the external electric field, and the trapped charge at the same polarity formed an electric field inside the sample, inhibiting the injection of more charge into the sample. However, the nano-zeolite in the XLPE/5wt% ZSM-5 composite was not modified and agglomerated together in the composite material, resulting in the production of large size defects, which weakened the effect of suppressing the space charge. At the same time, the XLPE/5wt% TAICS-ZSM-5-HBP and XLPE/7wt% TAICS-ZSM-5-HBP composites had better inhibition effects on space charge injections, as shown in Figure 6c,d. This was due to the introduction of TAICS-ZSM-5-HBP modified particles with the porous structure of nano-zeolite into the XLPE composite; the specific surface area of the particles was greatly increased, resulting in a larger interface area. At the same time, the modified grafting of TAICS-ZSM-5-HBP caused the particles to disperse uniformly in the composite, forming more deep traps. Thus, more carriers were captured under a high electric field, and a shielding layer with a greater potential energy was formed, which inhibited the external space charge injection. The results showed that the XLPE/5wt% TAICS-ZSM-5-HBP composite had the best inhibition effect. However, high-concentration doping can lead to a new agglomeration phenomenon, which is not conducive to the suppression of space charge in the XLPE/7wt% TAICS-ZSM-5-HBP composite. At 80 °C, the charge injection phenomenon was intensified with an increase in temperature. At a high electric field of 40 kV/mm, it can be seen from Figure 7a that a large amount of charge injection occurred in XLPE at the beginning of polarization. The charge density increased with an increase in the polarization time at the anode. However, the space charge in the XLPE/5wt%ZSM-5 composite material was significantly inhibited, and the maximum charge density at the anode dropped from close to 25 C/m^3^ to less than 15 C/m^3^, as shown in Figure 7b. As can be seen from Figure 7c, the XLPE/5wt% TAICS-ZSM-5-HBP composite also showed a good space charge inhibition effect at 80 °C, and no heteropolar charges appeared in the sample. The internal space charge density decreased significantly, which effectively inhibited the injection of space charge.

**Figure 8:**
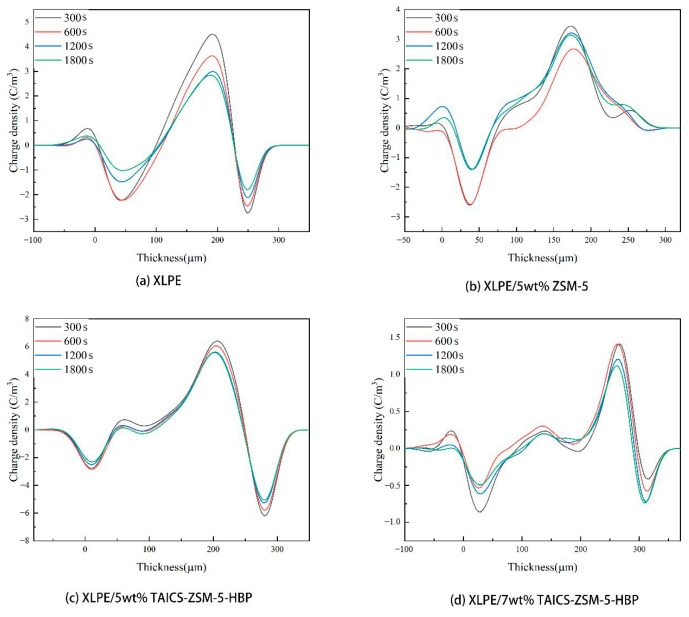
Space charge distribution of XLPE and composites during short circuit at 25 °C.

**Figure 9:**
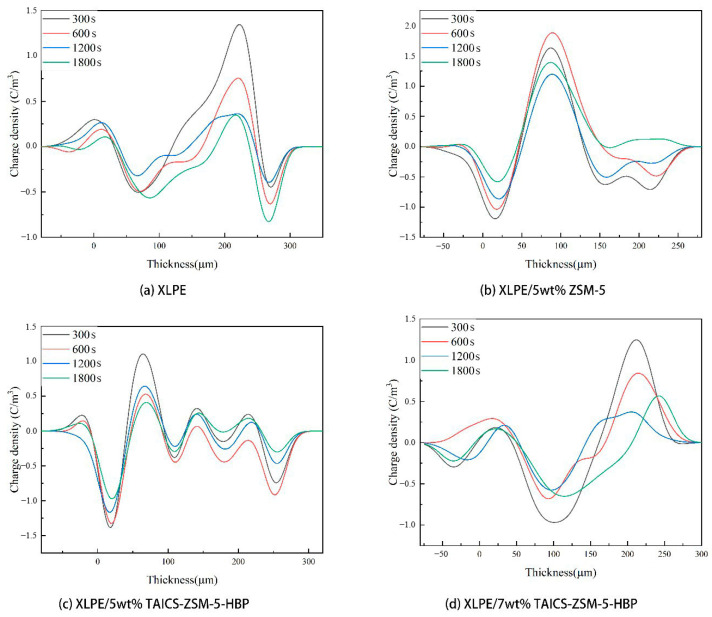
Space charge distribution of XLPE and composites during short circuit at 80 °C.

The space charge distribution of the XLPE material, XLPE/5wt% ZSM-5 composite, XLPE/5wt% TAICS-ZSM-5-HBP composite, and XLPE/7wt% TAICS-ZSM-5-HBP at 25 °C and 80 °C is shown in Figure 8 and Figure 9.

From Figure 8, it can be determined that the space charge dissipation rate of the XLPE material at 25 °C was obviously faster than that of the nanocomposite, and more charge was still stored in the nanocomposite after an 1800 s short circuit, among which, the XLPE/5wt% TAICS-ZSM-5-HBP composite had the lowest internal charge dissipation rate, and there were still some residual charges after the 1800 s discharge. This was due to the introduction of ZSM-5 zeolite as a porous material to produce a large number of traps in the composite material and increase the trap energy level. The modified TAICS-ZSM-5-HBP particles were connected to the three-dimensional network of XLPE, which dispersed the trap and provided a larger specific surface area, making it difficult for trapped electrons to break free from the trap, and there were still more charges left in the material after the short circuit. At 80 °C, the charge dissipation of the proximity electrode was faster, the middle charge dissipation was slower, the charge dissipation rate of the XLPE/5wt% TAICS-ZSM-5-HBP composite was still low when short-circuited, and it still had a good charge-binding effect at high temperatures. The above results are also consistent with the nanoparticle dispersion shown in Figure 4. The better the space charge inhibition effect of the sample, the better the dispersion of nano-zeolite. Meanwhile, the slower the space charge dissipation during the short circuit, the more deep traps existed, and the better the dispersion of nano-zeolite.

**Figure 10:**
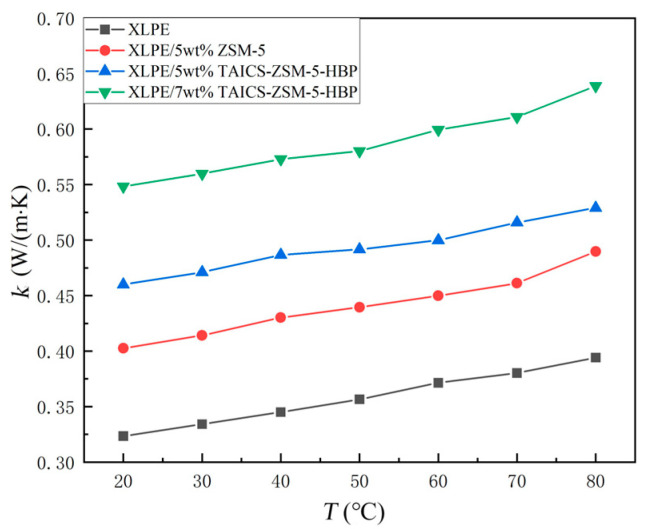
Relationship between thermal conductivity and temperature of XLPE and its zeolite composites.

The thermal conductivity of the XLPE materials, XLPE/ZSM-5 composites, and XLPE/TAICS-ZSM-5-HBP composites at different temperatures is shown in Figure 10. Since the glass transition temperature (Tg) of polyethylene is about −120 °C, its melting temperature, Tm, is about 110 °C. Moreover, XLPE is a thermosetting material, there is no clear Tg, and its melting temperature, Tm, is greater than 180 °C; no physical processes such as glass transition or melting will occur in the thermal conductivity test range. It can be seen from the figure that the XLPE materials, XLPE/ZSM-5 composites, and different concentrations of XLPE/TAICS-ZSM-5-HBP composites showed an approximate linear increase trend with an increase in the temperature. The introduction of TAICS-ZSM-5-HBP modified zeolite particles improved the dispersion of the modified particles in XLPE and significantly improved the thermal conductivity of the XLPE composites.

The overall thermal conductivity of the doped composites was higher than that of the XLPE materials, the thermal conductivity of the XLPE/TAICS-ZSM-5-HBP composites was superior to that of the XLPE/ZSM-5 composites, and the thermal conductivity of the composites with higher mass fractions was higher. At 20 °C, the thermal conductivity of the XLPE/5wt% TAICS-ZSM-5-HBP composites increased by 14.89% compared with the XLPE/5wt% ZSM-5 composites, and by 42.21% compared with the XLPE materials. Meanwhile, the XLPE/7wt% TAICS-ZSM-5-HBP composites increased by 69.59% compared with the XLPE materials. At 80 °C, the thermal conductivity of the XLPE/5wt% TAICS-ZSM-5-HBP composites increased by 11.89% and 34.27% compared with the XLPE materials. The XLPE/7wt% TAICS-ZSM-5-HBP composites increased by 62.83% compared with the XLPE materials.

There were both crystalline and amorphous regions in the polymer, and the crystalline part was more conducive to the propagation of phonons, which is related to the thermal conductivity. Therefore, a higher crystallinity was conducive to an improvement in thermal conductivity. In order to further verify the thermal conductivity, the crystallinity of the sample was measured using a differential scanning calorimeter (DSC), and the results are shown in Table 1. As can be seen from the table, the crystallinity of XLPE and XLPE/5wt% ZSM-5 was basically the same, which indicates that nano-zeolites without surface modification were agglomerated together, resulting in a large voidage and defects in the composite material. The crystallinity is difficult to improve, so that the thermal conductivity of the composite cannot be effectively improved. On the other hand, the crystallinity of the XLPE/TAICS-ZSM-5-HBP composite was significantly improved, so that the proportion of ordered crystal regions was larger, which effectively enhanced the phonon propagation and improved the thermal conductivity.

The HBP group of the rigid backbone structure with high thermal conductivity characteristics was introduced into the three-dimensional network structure of XLPE by TAICS-ZSM-5-HBP modified zeolite particles. It was used as the thermal conduction node of the TAICS-ZSM-5-HBP modified particles with the XLPE molecular chain and intermolecular chain heat conduction system, which not only increased the number of thermal conduction nodes, but also reduced the thermal resistance of the thermal conduction path. Moreover, the surface of the TAICS-ZSM-5-HBP modified zeolite particles was modified and grafted with HBP, which reduced the thermal resistance between the TAICS-ZSM-5-HBP modified zeolite particles and the XLPE molecular chain, and improved the thermal conductivity of the composite.

ZSM-5 zeolite, with a high thermal conductivity, was introduced into the XLPE matrix, and the particles and XLPE overlapped each other to form a new heat conduction path, so that the thermal conductivity of the XLPE/ZSM-5 composite was higher than that of the XLPE material; the TAICS-ZSM-5-HBP modified zeolite particles were more uniformly dispersed in the XLPE matrix than the ZSM-5 zeolite particles, and the TAICS-ZSM-5-HBP modified zeolite particles had a lower thermal resistance to forming a new heat conduction path in the XLPE matrix, which significantly improved the thermal conductivity of the composite.

## 4. Conclusions

This paper aimed to improve the thermal conductivity and insulation properties of crosslinked polyethylene for HVDC cables. Modified zeolite (TAICS-ZSM-5-HBP) with an auxiliary crosslinking agent function and grafted hyper-arylamide on the surface was prepared. A composite material (XLPE/TAICS-ZSM-5-HBP) was prepared by mixing, hot melting, and chemical crosslinking. Its thermal conductivity and space charge inhibition performance were studied and analyzed using experimental tests, and the following conclusions were mainly drawn:

The surface modification and grafting of nano-zeolite effectively improved the dispersion of nanoparticles in the polymer matrix, the nano-zeolite was uniformly dispersed in the prepared nanocomposites, and a three-dimensional network structure was formed through the crosslinking reaction.

The space charge distribution tests showed that the space charge accumulation phenomenon of XLPE/TAICS-ZSM-5-HBP was significantly improved compared with that of XLPE, and the charge dissipation was significantly controlled after the introduction of TAICS-ZSM-5-HBP modified particles. Nano-zeolite doping produces more traps in the composite material and increases the trap energy level.

The thermal conductivity test results showed that TAICS-ZSM-5-HBP-modified-particle doping can effectively improve the thermal conductivity of the nanocomposites. The HBP group of the rigid backbone structure, with high thermal conductivity characteristics, was introduced into the three-dimensional network structure of XLPE using TAICS-ZSM-5-HBP modified zeolite particles.

## Figures and Tables

**Table 1:** Crystallinity of the XLPE and its zeolite composites.

Sample Name	XLPE	XLPE/5wt% ZSM-5	XLPE/5wt% TAICS-ZSM-5-HBP	XLPE/7wt% TAICS-ZSM-5-HBP
Crystallinity	30.93%	30.65%	34.77%	35.93%

## Data Availability

Data are contained within the article.
